# *N*-Acetylcysteine Reverses Monocrotophos Exposure-Induced Hepatic Oxidative Damage via Mitigating Apoptosis, Inflammation and Structural Changes in Rats

**DOI:** 10.3390/antiox11010090

**Published:** 2021-12-30

**Authors:** Jagjeet Singh, Annu Phogat, Chandra Prakash, Sunil Kumar Chhikara, Sandeep Singh, Vinay Malik, Vijay Kumar

**Affiliations:** 1 Department of Zoology, Maharshi Dayanand University, Rohtak 124001, India; jagjeet.rs.zoo@mdurohtak.ac.in (J.S.); annu.rs.zoo@mdurohtak.ac.in (A.P.); vinaymalikzoo@mdurohtak.ac.in (V.M.); 2 Neurobiology Laboratory, School of Life Sciences, Jawaharlal Nehru University, New Delhi 110067, India; chandrabt87.rs.biochem@mdurohtak.ac.in; 3 University Institute of Engineering and Technology, Maharshi Dayanand University, Rohtak 124001, India; sunilchhikara.uiet@mdurohtak.ac.in; 4 Department of Biochemistry, Maharshi Dayanand University, Rohtak 124001, India; ssingh.biochem@mdurohtak.ac.in

**Keywords:** *N*-acetylcysteine, hepatic oxidative stress, monocrotophos, antioxidants, apoptosis, inflammation

## Abstract

Oxidative stress-mediated tissue damage is primarily involved in hepatic injuries and dysfunctioning. Natural antioxidants have been shown to exert hepatoprotective, anti-inflammatory and antiapoptotic properties. The present study evaluated the effect of *N*-acetylcysteine (NAC) against monocrotophos (MCP) exposure-induced toxicity in the rat liver. Albino Wistar rats were divided into four groups: (1) control, (2) NAC-treated, (3) MCP-exposure, (4) NAC and MCP-coexposure group. The dose of MCP (0.9 mg/kg b.wt) and NAC (200 mg/kg b.wt) were administered orally for 28 days. Exposure to MCP caused a significant increase in lipid peroxidation, protein oxidation and decreased glutathione content along with the depletion of antioxidant enzyme activities. Further MCP exposure increased pro-inflammatory cytokines levels and upregulated Bax and Caspase-3 expressions. MCP exposure also caused an array of structural alternations in liver tissue, as depicted by the histological and electron microscopic analysis. Thepretreatment of NAC improved glutathione content, restored antioxidant enzyme activities, prevented oxidation of lipids and proteins, decreased pro-inflammatory cytokines levels and normalized apoptotic protein expression. Treatment of NAC also prevented histological and ultrastructural alternations. Thus, the study represents the therapeutic efficacy and antioxidant potential of NAC against MCP exposure in the rat liver.

## 1. Introduction

Monocrotophos (MCP) is an insecticide used to control sucking and chewing pests of fruits and vegetables. In India, it is labeled a “restricted use” insecticide because it is banned for use on vegetables due to its high persistence, but it is allowed to be used on commercial crops. Despite its restricted-for-use label, it is used extensively in various crops, such as rice, cotton, soybeans, sugarcane, maize, groundnut, etc., owing to its high effectiveness and low cost [1,2]. Due to its widespread use, MCP possesses major environmental risk factors for nematodes, arthropods, fish and mammals, including humans. Various reports have confirmed the presence of MCP in groundwater, soil and in agricultural products, indicating its easy entry in the food chain, which may result in environmental contamination [3,4,5]. A study has shown 0.75 mg MCP/kg in soil after pesticide spray in agriculture fields [6]. The persistence of MCP in the environment and edible items makes humans susceptible to its long-term exposure, even if the contamination is below the acceptable limits. In relation to this, a study by Kumari et al. [7] demonstrated that MCP is unintentionally consumed above the maximum daily limit of MCP (6 μg/kg b.wt per day). Both chronic and acute exposures to MCP and its residues is known to induce hepatotoxicity [8], nephrotoxicity [9], neurotoxicity [10] and genotoxicity [11] in mammals. 

Pesticides account for more than 200,000 deaths per year globally due to either intentional and acute intoxication [12]. Despite the fact that acute exposure is a major reason for mortality, chronic exposure is considered a major reason for the morbidity of pesticide exposure in agricultural workers and consumers. A study involving 214 farmers associated with the spraying of MCP and other pesticides reported a two- to five-fold increase in symptoms of pesticide toxicity [13]. The application of MCP and other pesticides in paddy fields caused an increase in mortality rate by 27% in the Philippines population [14]. Similarly, it was reported that MCP exposure was responsible for 22.2% of mortalities associated with pesticide poising in Brazil. It is estimated that in India, MCP is majorly used and its exposure is involved in the maximum numbers of mortality by poisoning [15,16].The liver has long been considered as a major target organ of MCP-induced toxicity [17,18]. Studies have demonstrated that MCP exposure induces morphological and physiological changes in the liver [17,19]. The generation of reactive oxygen species (ROS) and antioxidant depletion are key factors for MCP-induced hepatotoxicity. In fact, MCP exposure has been reported to alter the enzymatic as well as non-enzymatic antioxidants in the liver cells, suggesting oxidative stress generation. The combined effects of cholinergic and oxidative stress cause structural anomalies and physiological and developmental alterations via cellular damages and apoptosis [20]. Although the mechanism of liver injuries is very complex, it has been proved that apoptosis and inflammation are closely associated and play a critical role in the oxidative stress-mediated hepatic dysfunctions. The generation of ROS can regulate a number of signaling pathways and mediator molecules inside hepatic cells. Among them, Bax—a pro-apoptotic factor and Bcl-2—an anti-apoptotic factor, play a significant role in oxidative stress-induced cellular injuries via caspases-3 activation. It was demonstrated that pro-inflammatory cytokines trigger an inflammatory cascade and apoptosis in hepatocytes and nephrocytes [21,22]. Apoptosis might induce inflammation and vice versa in liver tissue via the disruption of hepatocyte integrity, deposition of apoptotic bodies and induction of necrotic factors [23]. In relation to this, MCP exposure-mediated oxidative damage was demonstrated to be associated with inflammatory damage and multiple organ disorders in rabbits [24].

Antioxidants are biomolecules capable of activating the response of a biological system to reduce the toxic effects of xenobiotics. Natural antioxidants primarily consist of active groups, such as thiols, polyphenols and isoquinolines, etc. These compounds are assumed to protect the biological system from oxidative stress by scavenging free radicals resulting from pesticides exposure [25]. *N*-acetylcysteine (NAC) is a thiol-containing reducing agent that possesses many pharmacological properties, such as anti-oxidative, anti-apoptotic, anti-inflammatory and anti-neoplastic [26,27,28,29,30]. It is a potent neurostimulator and is widely recognized for its effectiveness against neurodegenerative disorders [31,32] and psychiatric disorders [33,34]. Along with its organ-specific therapeutics applications, NAC is effective against metal toxicity [35,36,37], pesticide-induced hepato-renal toxicity, neurotoxicity and reproductive toxicity [38,39,40] and other health conditions, including infectious diseases [41], infertility [42,43,44] and hepatic diseases [45]. Many studies have evaluated the effects of some antioxidants against MCP toxicity in aquatic animals. However, very few studies have focused on the toxic effects of MCP exposure in mammals and its amelioration through antioxidants and plant extracts. *N*-acetylcysteine is a broad-spectrum biomolecule that easily gets incorporated inside tissues with higher in vivo stability. As per the literature survey, no study has evaluated the effects of NAC against MCP-induced toxicity in rodents. Hence, the present study is executed to evaluate the effects of NAC on biochemical and structural alterations in rat liver following MCP exposure.

## 2. Materials and Methods

### 2.1. Chemicals

Monocrotophos (Cat no. 36173), NAC (Cat no. A7260), acetylthiocholine (ATC) (Cat no. A5751), bovine serum albumin (BSA) (Cat no. A3059), triton-X-100 (Cat no. T8787), guanidine-HCl (Cat no. 50950), H_2_O_2_ (Cat no.1086001000), hydroxylamine-HCl (Cat no. V800215) and potassium bromide (KBr) (Cat no. 221864) were purchased from Sigma–Aldrich, St Louis, MO, USA. 5,5-dithiobis-2-nitrobenzoic (DTNB), nitro blue tetrazolium (NBT), thiobarbituric acid (TBA), ethylene diamine tetraacetic acid (EDTA), 2,4-dinitrophenylhydrazine (DNPH) and trichloroacetic acid (TCA) were purchased from Sisco Research Laboratory, Mumbai, India. Primary antibodies against Bax (Sc-493), Bcl-2 (Sc-783), Caspase-3 (Sc-7148), β-actin (Sc-4778) and HRP labeled secondary antibodies were purchased from Santa Cruz Biotechnology, Santa Cruz, CA, USA. AmershamProtran Nitrocellulose membrane (No-10600008) was purchased from GE healthcare Life Sciences, Germany. Glassware and plastic ware used in the study were autoclaved and sterilized before use.

### 2.2. Animals and Their Care

Albino rats of the Wistar strain weighing 150–180 g were kept in the Central Animal House Facility of Maharshi Dayanand University under standard conditions (temperature 25 ± 1 °C, relative humidity 50 ± 10%; 12 h alternate light-dark cycle)in polypropylene cages with free access to water and standard rodent laboratory feed pellets. All protocols involving animals were followed according to the guidelines of the Committee for Control and Supervision of Experiments on Animals, India. Permission to use animals was duly approved by the Institutional Animal Ethical Committee. 

### 2.3. Experimental Design

- A total of 20 rats were randomly assigned to the following four groups containing 5 rats in each group: Control group (Cont): Rats received 1 mL of distilled water (vehicle), intragastrically, for 28 days.
- N-acetylcysteine Treated Group: Rats received 1 mL of NAC (200 mg/kg b.wt) dissolved in distilled water, intragastrically, for 28 days.
- Monocrotophos Exposure Group: Rats received MCP (0.9 mg/kg b.wt) dissolved in distilled water, intragastrically, for 28 days.
- N-acetylcysteine + MCP Coexposure Group: Rats received MCP and NAC dissolved in distilled water, intragastrically, for 28 days. In this group, NAC was given 2 h before MCP administration.

The dose and time of NAC treatment were based on previous studies that have shown the selected dose significantly effective against stress conditions [46,47]. The dose of MCP was selected based on previous studies that have reported the selected dose to cause hepatic toxicity in rats [48]. For the MCP dose, 100 mg MCP was dissolved in 2 mL distilled water to prepare a stock solution. An amount of 200 μL of MCP stock solution was dissolved in distilled water to make a 70 mL working dose. An amount of 2.5 g NAC was dissolved in distilled water to make the final volume of 75 mL. The working dose was prepared every week.

Rats were euthanized via carbon dioxide asphyxiation after the last dose administration. Liver tissue was dissected, washed in 0.9% ice-cold normal saline and used for biochemical, Fourier transform infrared spectroscopy (FTIR), histopathology and electron microscopy analysis.

### 2.4. Tissue Homogenate Preparation

A portion of liver tissue was added with a homogenizing buffer (pH 7.4), containing sucrose (0.25 M), EDTA (1 mM) and tris (5 mM). A 10% tissue homogenate was prepared using a motor-driven homogenizer (Perfit, Gupta Scientific Industries, Ambala, India) and centrifuged at 4 °C at 5000 rpm for 10 min. The resulting supernatant was stored in fresh tubes and used for biochemical assays. The protein content in each sample was determined using BSA as standard [49].

### 2.5. Acetylcholinesterase (AChE) Activity Assay

Acetylcholinesterase activity in serum and liver tissue homogenate was estimated using the method of Ellman et al. [50]. Briefly, 0.1 mL of tissue homogenate was added to the reaction tube containing 2.4 mL sodium phosphate buffer (0.1 M), 0.2 mL DTNB (10 mM), 0.2 mL Triton X-100 (0.013%) and 0.2 mL ATC (10 mM); the resulting reaction mixture was read on UV-Vis spectrophotometer (Shimadzu 1900, Duisburg, Germany) at 412 nm for 2 min. Results were expressed as nmol product formed/min/mg protein. 

### 2.6. Estimation of Lipid Peroxidation (LPO)

Lipid peroxidation was estimated by the method of Wills [51]. Briefly, 1 mL of 10% TCA was added to the liver homogenate and centrifuged to precipitate the proteins. The supernatant was mixed with 1.5 mL TBA (0.67%) and placed in a boiling water bath to develop the color. The amount of thiobarbituric acid reactive substances formed was determined and used to express the peroxidation of lipids as nmol MDA/mg protein.

### 2.7. Estimation of Protein Oxidation

The protein carbonyl in liver tissue homogenate was quantified after derivatization with DNPH [52]. Briefly, 1 mL of TCA (10%) was added to 0.2 mL tissue homogenate to precipitate the proteins. An amount of 0.5 mL DNPH (10 mM) was added and allowed to incubate for 1 h at room temperature. Then, 0.5 mL of TCA (20%) was added and centrifuged at 11,000× *g* for 3 min. The obtained pellets were subsequently washed thrice with 1:1 ethanol/ethyl acetate solution and resuspended in 1 mL guanidine-HCl (6 M). The insoluble fraction was removed by centrifugation and the absorbance of the supernatant was recorded at 370 nm. The protein carbonyl content was expressed as nmol carbonyls/mg protein. 

### 2.8. Antioxidant Enzyme Assays

Catalase (CAT) activity in liver homogenate was assayed by monitoring the enzyme-catalyzed degradation of H_2_O_2_ at 240 nm for 3 min [53]. Briefly, the reaction mixture was prepared by mixing 1.9 mL potassium phosphate buffer (0.925 mM) and 1 mL H_2_O_2_ (10 mM) in 0.1 mL liver homogenate. The decrease in absorption was recorded at 240 nm and the amount of H_2_O_2_ decomposed was calculated. Results were expressed as μmol H_2_O_2_decomposed/min/mg protein.

Superoxide dismutase (SOD) activity in liver homogenate was determined by measuring the NBT reduction [54]. Reaction mixture was prepared by mixing 1.9 mL sodium carbonate buffer (50 mM), 0.75 mL NBT (96 μM), 0.15 mL triton-X-100 (0.6%), 0.15 mL hydroxylamine hydrochloride (2 mM) and 0.1 mL liver homogenate. The reaction mixture was read at 560 nm for 3 min. Results were expressed as U/mg protein.

### 2.9. Glutathione Estimation (GSH)

Glutathione content in liver tissue homogenate was estimated using DTNB as a substrate [55]. An amount of 0.2 mL liver homogenate was mixed with 0.4 mL EDTA (0.02 M) followed by 10 min incubation on an ice bath. After incubation, 4 mL distilled water and 1 mL TCA (10%) were added to the mixture, incubated on ice for 10 min followed by centrifugation at 3000 rpm for 15 min. Then, 2 mL of supernatant was taken and mixed with 4 mL tris buffer (0.4 M) and 0.1 mL DTNB (0.01 M). The mixture was read immediately at 412 nm and the results were expressed as nmol/mg protein.

### 2.10. Gene Expression Analysis of Inflammatory Cytokines

Total RNA was isolated from liver tissue using RNAsureMinikit (Nucleopore). RNA was reverse-transcripted into 20 μL cDNA using the RevertAid First Strand cDNA Synthesis Kit (Invitrogen, Thermofisher Scientific, Vilnius, Lithuania). cDNA products were subjected to semiquantitative RT-PCR analysis on a gradient thermal cycler (PEQ LAB, Erlangen, Germany). The primers sequence used for the studied genes is shown in Table 1.

### 2.11. Western Blot Analysis

Liver tissue was homogenized with lysis buffer and centrifuged at 10,000× *g* for 15 min and the supernatant was collected. The expression analysis for apoptotic proteins was done using the respective primary and secondary antibodies, as described earlier [56]. 

### 2.12. Fourier Transforms Infrared Analysis

Liver tissue samples were prepared for FTIR analysis as described earlier by Akkas et al. [57]. Briefly, liver tissues were lyophilized overnight and mixed by grinding with KBr in 1:100 ratios (*w/w*). After proper mixing, the powder was pressed under hydraulic press at 1100 kg/cm^2^ pressure for 2 min to form smooth-surfaced uniform pellets. The spectra were recorded in the region 4000–400 cm^−1^ on an FTIR spectrometer (Alpha, Bruker Optics, Ettlingen, Germany). The obtained spectra were further analyzed in two separate regions (3800–2700 cm^−1^ and 1800–1400 cm^−1^) using ORIGIN^®^ 19 software (Origin Lab Corporation, Northampton, MA, USA)

### 2.13. Histolopathological Study 

For histological analysis, dissected liver tissues were stored in 70% ethanol after 24 h of fixation in formalin. Stored tissues were dehydrated in different grades of alcohol (30%, 50%, 70%, 90% and absolute). After dehydration, the fixed tissues were embedded in paraffin wax after saturating with xylene and wax. Tissue sections (5 µm thickness) were cut using a microtome and fixed on slides using egg albumin and heat. Slides were dipped in xylene to deparaffinize the tissue sections and then processed for haematoxylin, eosin and Van Geison staining. The stained slides were mounted with dibutylphthalatepolystyrene xylene and cover-slipped. Tissue sections were observed under the microscope (Nikon Eclipse Ci-L, Tokyo, Japan) and were photographed. The histological lesions and severity of lesions observed were scored using the ordinal scale as none (−), mild (+), moderate (++) and severe (+++) damage following Chakroun et al. [58].

### 2.14. Electron Microscopy

For electron microscopy tissues of the liver (1–2 mm^3^) were fixed in 2.5% glutaraldehyde and post-fixation storage was done in phosphate buffer at room temperature. After primary fixation, tissues were fixed in osmium tetraoxide and were embedded in epoxy resin after graded dehydration in ethanol. Then, the epoxy resins were dried at 55°C for 48 h and cut into thin sections using ultra-microtome. The prepared sections were stained and examined under the transmission electron microscope (Tecnai TF-30, FEI-Thermo Fisher) and images were captured.

### 2.15. Statistical Analysis 

Data were analyzed for statistical significance by one-way analysis of variance (ANOVA) followed by Tukey’s post hoc test. Results with values for *p* < 0.05 were considered statistically significant.

## 3. Results

### 3.1. Acetylcholine Esterase Activity

**Figure 1:**
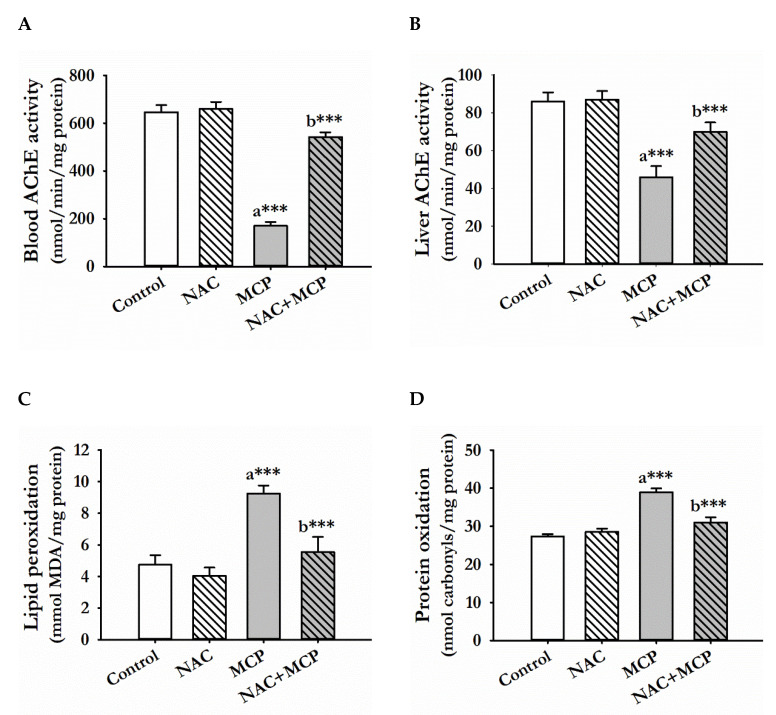
NAC pre-treatment restored AChE activity and prevented oxidative stress in the liver of MCP-exposed rats. (**A**,**B**) Enzymatic activity of AChE in the serum and liver tissue, (**C**) levels of MDA and (**D**) carbonyl content in the liver tissue of the different experimental groups. The values represented are mean ± S.D. (N = 5). ^a^ as compared to the control group; ^b^ as compared to MCP-exposed group. *** *p* < 0.001, significantly different.

There was a significant depletion of AChE activities in serum and liver tissue in the rats exposed to MCP insecticide as compared to control. Prior treatment of NAC with MCP for 28 days significantly elevated both serum and liver AChE activities as compared to MCP administrated rats, while the NAC-alone-treated rats showed no significant change in AChE activity, both in serum and liver tissues, compared to the control rats (Figure 1A,B).

### 3.2. Lipid Peroxidation

Monocrotophos exposure for 28 days significantly caused the peroxidation of lipids in liver tissues of rats as compared to the control group. Administration of NAC to the MCP-exposed group significantly prevented LPO as compared to the MCP-exposed rats. The *N*-acetylcysteine alone treatment showed no significant change in LPO of liver tissue as compared with the control rats (Figure 1C).

### 3.3. Protein Oxidation

Protein oxidation was significantly increased in the liver tissue of rats administered with MCP for 28 days. In contrast, prior administration of NAC to MCP-exposed animals significantly prevented protein oxidation as compared to the MCP-intoxicated rats, while treatment of NAC alone showed no significant change in protein oxidation as compared to the control rats (Figure 1D).

### 3.4. Antioxidant Enzyme Activities

Monocrotophos exposure for 28 days significantly decreased the activities of SOD and CAT as compared to the control rats. Treatment of NAC to MCP-intoxicated rats significantly restored both SOD and CAT activities as compared to the MCP-alone-exposed rats. Administration of NAC alone showed no significant changes in antioxidant enzyme activities as compared to the control rats (Table 2).

### 3.5. Glutathione Content

Monocrotophos exposure for 28 days caused significant depletion of GSH content as compared to control rats. Treatment of NAC before MCP exposure significantly restored GSH content as compared to MCP-exposed rats. The control- and NAC-alone-administered rats showed comparable GSH content (Table 2).

### 3.6. Evaluation of Pro-Inflammatory Cytokines

**Figure 2:**
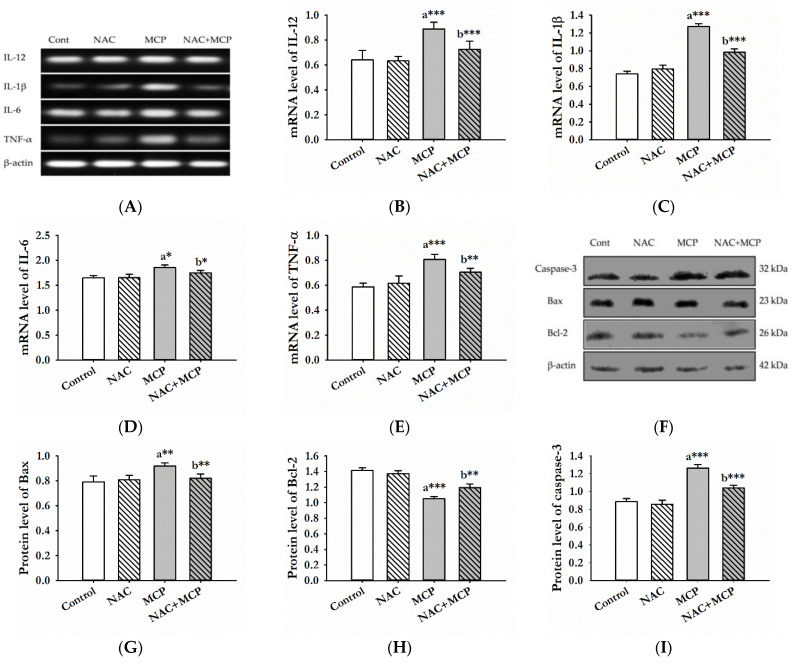
NAC pre-treatment attenuates inflammation and apoptosis in the liver of MCP-exposed rats. (**A**) Representative image of mRNA expression of IL-12, IL-1β, IL-6 and TNF-α. (**B**–**E**) Relative mRNA level of different pro-inflammatory markers with respect to β-actin. (**F**) Western blotting showing the changes in protein expressions of Bax, Bcl-2 and caspase-3 in control and experimental groups. (**G**–**I**) Relative protein levels of Bax, Bcl-2 and caspases-3 with respect to β-actin. The values represented are the values represented are mean ± S.D. (N = 3). ^a^ as compared to the control group; ^b^ as compared to MCP-exposed group. * *p* < 0.05, ** *p* < 0.01, *** *p* < 0.001 significantly different.

To investigate the effects of NAC and MCP exposure on the mRNA expression of inflammatory markers, a PCR analysis of IL-1β, IL-6, IL-12 and TNF-α was done. MCP exposure for 28 days caused significant increase in the mRNA levels of IL-1β, IL-6 and IL-12 compared to the control group. Similarly, TNF-α expression was also increased significantly in the liver tissue of the MCP-exposed rats. Furthermore, pre-treatment with NAC significantly decreased the mRNA expression of IL-1β, IL-6, IL-12 and TNF-α. NAC-alone-administration did not cause any changes in the expression of these pro-inflammatory cytokines compared to control group (Figure 2A–E).

### 3.7. Western Blot Analysis

Further, to evaluate the effect of MCP exposure-induced oxidative stress on apoptotic proteins, we analyzed the expression of Bax, Bcl-2 and caspase-3. The expression of pro-apoptotic proteins, Bax and caspases-3 increased significantly, while the expression of Bcl-2 protein decreased in the liver tissue of rats following MCP exposure. However, NAC pretreatment to the MCP-exposed rats significantly alleviated the change in the expression of apoptotic proteins. There was no effect of the NAC alone treatment on expression levels of these proteins as compared to control rats (Figure 2F–I).

### 3.8. Fourier Transforms Infrared Analysis of Lipids and Proteins

**Figure 3:**
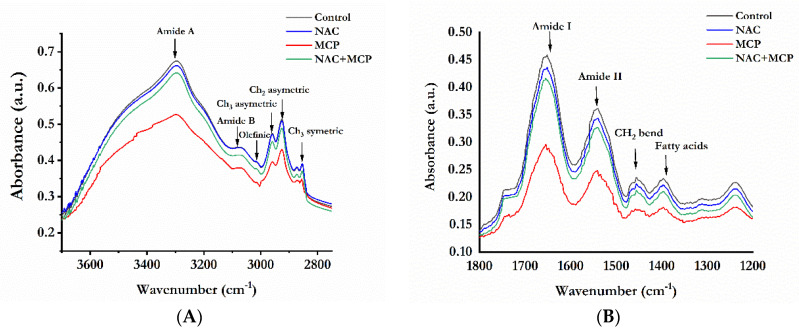
NAC pre-treatment restores MCP exposure-induced structural alterations in rat liver.The FTIR spectra are representing the selected wavenumber range of (**A**) region 3700 to 2750 cm^−1^; (**B**) region 1800 to 1200 cm^−1^. The spectra are representative of N = 3.

The FTIR absorption spectra between 4000 cm^−1^ to 400 cm^−1^ were analyzed for protein and lipid structure in the rat liver. A second derivative FTIR spectra was studied in two different regions, i.e., 3800–2750 cm^−1^and 1800–1400 cm^−1^ (Figure 3A,B). The peaks assignment showed changes in lipids and proteins among the control and MCP-exposed groups. As shown in Table 3, the peaks that appeared at 3297 and 3080 cm^−1^ arise from *n*-H and =CH stretching of proteins (amide A and B regions). Table 4 shows that there was a significant decrease in the band area of the *n*-H stretch of amide A and B following MCP exposure. The olefinic acid band observed at 3014 cm^−1^ that mainly arises due to C-H stretching on HC = CH groups was also decreased. The area of peaks assigned to CH_3_ asymmetric (~2959 cm^−1^), CH_2_ asymmetric (~2925 cm^−1^) and CH_2_ symmetric (~2854 cm^−1^) stretch of lipids was also reduced significantly in MCP-exposed rats (Table 4). Minor changes in peak positions were also observed for these regions. The treatment of NAC- to MCP-exposed rats restored the area of the peaks representing lipids and proteins structure in the region (3700–2750 cm^−1^) towards control values (Table 4).

In the 1800–1400 cm^−1^ region (Figure 3B), MCP exposure significantly altered the peak area of C = O stretch (~1652 cm^−1^) of amide A along with N-H and C-N stretch (~1541 cm^−1^) of amide B (Table 3). Treatment of MCP also caused a significant decrease in the peak area of C = O stretch (~1745 cm^−1^), -CH_2_ bend (~1456 cm^−1^) and COO- stretch (~1397 cm^−1^) of lipids and fatty acids (Table 4). Minor shifts in peak positions of these regions were also observed in the MCP exposure group (Table 3). Administration of NAC to MCP-exposed rats significantly prevented the structural changes corresponding to proteins and lipids, as evident from the restoration of altered peak position and area values (Table 4).

### 3.9. Histopathology Analysis

**Figure 4:**
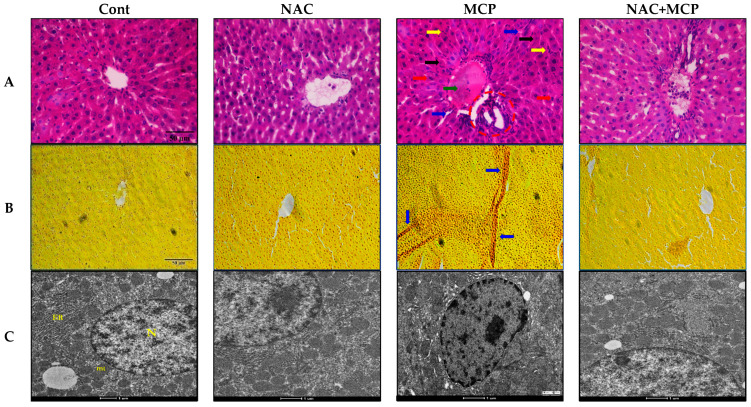
Photomicrograph of the liver tissue sections. (**A**) H and E staining (200*); circle indicates the area of inflammation and infiltrated cells; black arrows indicate pyknotic nuclei; blue arrows depict sinusoidal space; yellow arrows depict activated kuffer cells; green arrow indicates congestion of central vein; red arrows indicate apoptotic hepatocytes. (**B**) Van Geison’s staining (200*); arrows depict the fibrosis. (**C**) Transmission electron microscopy images; N—nucleus, ER—endoplasmic reticulum, mt—mitochondria.

Light microscopy of the hematoxylin and eosin-stained liver tissue of control rats exhibited normal architecture of bile duct, portal triads and hepatocyte. Hexagonal hepatocytes were arranged radiating from the central vein, separated by blood sinusoids with uniform cytoplasm. Monocrotophos exposure caused congestion of the central vein, sinusoidal disruption, necrosis and cell infiltration. Pyknotic nuclei and apoptotic hepatocytes were also observed in MCP-exposed rat liver sections. In contrast, pre-treatment of NAC to MCP-exposed rats effectively improved the histo-architecture of hepatocytes as evident by the reduction of necrosis and apoptotic damage. The *N*-acetylcysteine-alone-treated group showed normal histological architecture similar to the controls (Figure 4A and Table 5).

Van Geison’s stain is used for differential staining connective tissues and detects fibrosis in the tumor and other pathological conditions. Van Geison’s staining of the liver sections of rats exposed to MCP exhibited significant fibrosis in liver tissues. Pre-treatment of NAC to this group effectively prevented liver fibrosis. Control- and NAC-alone-treated rats showed normal connective tissue arrangements in hepatic tissue sections (Figure 4B and Table 5).

### 3.10. Transmission Electron Microscopy

Ultrastructural investigation of MCP-exposed liver tissue sections showed marked alterations in nuclear structure as compared to the control tissue sections. Change in nucleus shape, chromatin condensation and break in the nuclear envelope were observed as major alterations in MCP-exposed rats. Conversely, pre-treatment of NAC to MCP-exposed rats maintained the shape of nucleus and intact nuclear envelope as compared to MCP-exposed rats. The control and NAC-treated groups showed normal shape and size of the nucleus and nuclear envelope (Figure 4C and Table 5).

## 4. Discussions

Monocrotophos exposure is known to induce oxidative stress, reduction of antioxidant levels and causes apoptosis that could lead to multi-organ failure and severe genetic alterations [10,59,60]. Accumulating evidence from recent studies has suggested the liver as the main targeted organ for pesticide-induced toxicity [61,62,63]. Depletion of endogenous antioxidants and biomolecular damages are common and the most negative aspects of pesticide exposure-induced hepatotoxicity. Several natural supplements are known to attenuate oxidative injuries induced by pesticide exposure. Antioxidative, anti-inflammatory and potential to act as a precursor for endogenous antioxidants are pre-requisites for these protective supplements [25]. *N*-acetylcysteine is one such natural supplement that functions as the precursor to endogenous GSH synthesis and acts as a nutritional supplement as well as an antidote for GSH deficiency in toxicity, diseases and metabolic disorders [38]. It is known to protect paraquat acute poisoning [64], deltamethrin-mediated in vitro cytotoxicity [65] and fipronil-induced hepatic injuries effectively [38]. Based on the strong antioxidant potential of NAC against pesticide toxicity, we have evaluated the antioxidant potential of NAC against the MCP-induced hepatotoxicity in rats. The findings of the study indicated that pretreatment of NAC for 28 days ameliorated oxidative stress and restored cellular antioxidants by regulating enzymatic activities in hepatic tissue, and it has also prevented tissue damage.

Monocrotophos is a well-known AChE inhibitor and causes cholinergic toxicity. The present study demonstrated that MCP exposure caused a decline in AChE activity in the serum and liver tissue of rats. Inside the body, MCP is readily transformed into reactive metabolites, which block the esteratic site of AChE and inhibit its activity [66]. Previous studies have also observed AChE inhibition in mammalian tissues following MCP exposure [66,67]. The present study supports the anticholinergic effect of NAC against MCP exposure in mammals. Treatment of NAC to MCP exposure recovered AChE levels in both liver and serum. Our results are in agreement with the findings of previous studies that have reported that NAC treatment recovers AChE activity in pesticide-induced toxicity in rats [68,69]. 

Oxidative stress has been depicted as a central mechanism of hepatotoxicity following xenobiotics exposure and arises when the critical balance between oxidants and antioxidants is disrupted inside the body [70,71,72]. The disruption between pro-oxidants and antioxidants could be attributed to free radical accumulation, antioxidants depletion, or both [73,74]. Furthermore, the generation of oxidative stress leads to an attack on lipids and proteins inside cells. Quantification of LPO and protein oxidation is a reliable tool to investigate oxidative stress-mediated toxicity [75,76]. In the present study, MCP exposure for 28 days caused LPO and protein oxidation in rat liver that was significantly reversed by prior administration of NAC following MCP exposure. The effects of the MCP-induced oxidative injuries to lipids and proteins were also evident from FTIR findings that indicated a possible change to the backbone of lipid and protein structure. A reduction in-band area and shift in band position of 3297 cm^−1^, 3080 cm^−1^, 1652 cm^−1^ and 1540 cm^−1^ is indicative of the structural rearrangement of proteins inside the cell. It is well known that the CH_2_ stretching band at 2854 cm^−1^ is an indicator of the “state of order” of the biological membrane. A decrease in band area value at 2854 cm^−1^ confirmed MCP-mediated alteration in biological membrane and mobility of fatty acids [77]. Further, changes in CH_2_ asymmetric and CH_2_ symmetric stretching of lipids also depicted conformational disorder and alteration in lipid acyl chain flexibility. The alteration in olefinic =CH band area value at 3014 cm^−1^ indicates the change in the number of unsaturated lipids [78]. The study observed that a decrease in the peak of amide I and II were consistent with the decline in amide A band area values. Any change in the position of amide bands or decrease in the peak area of amide bands may quantitatively reflect alterations in the composition of the protein secondary structure. This may be attributed to the increased protein oxidation and LPO, indicating a reduction in free radical scavengers by MCP exposure in rat liver, as evident from biochemical and histological studies. The administration of NAC along with MCP effectively ameliorated these structural and functional alterations in lipids and proteins. 

SOD and CAT are the main enzymes that assist in the elimination of oxidants formed during the bioactivation of pesticides [39]. These enzymes convert superoxide radicals and peroxides radicals into water and oxygen, thus removing the ROS formed during the bioactivation of xenobiotics [39,79]. We found a marked decrease in SOD and CAT activities in MCP-exposed rat liver, suggesting decreased scavenging capacity for free radicals. The depletion of cellular antioxidants in the liver following MCP exposure has also been reported earlier by various studies [17,19,80]. The *N*-acetylcysteine-mediated augmentation of CAT and SOD activities in MCP-exposed rats might indicate the free radical scavenging property of NAC. The results support various earlier in vitro studies demonstrating that NAC can directly scavenge free radicals [69,81,82]. 

Intracellular GSH is a thiol-containing non-enzymatic antioxidant and plays a pivotal role in the detoxification and metabolism of both endogenous and exogenous toxic substances [83]. It is an important cellular antioxidant and a biomarker of oxidative stress [84]. The present study shows that MCP exposure caused severe depletion in cellular GSH levels. The decline of GSH content is in concordance with various in vivo and in vitro studies that have implicated GSH depletion in MCP-induced toxicity [67,80,85]. We also observed that NAC treatment significantly increased GSH content in MCP-exposed rats. In fact, NAC is a source of cysteine that is a precursor of de novo GSH synthesis [86]. After the incorporation of NAC into cells, it is readily deacylated to deliver intact cysteine for GSH generation. Further NAC has direct antioxidant properties that might have quenched free radicals right at their formation and have protected GSH from overutilization. These results are in agreement with the findings of some earlier studies that have also observed restoration of cellular GSH levels against pesticide-induced oxidative stress in rat tissues following NAC supplements [47,87,88]. Increased LPO, protein oxidation and depressed antioxidants status in hepatic tissue following MCP exposure suggested that cytotoxic effects were imposed by oxidative stress in rats.

It has been demonstrated that inflammatory cytokines are involved in the progression of oxidative stress. The cytokines TNF-α, IL-1β, IL-6 and IL-12 are directly associated with free radical-mediated oxidative stress [89]. TNF-α is a prime pro-inflammatory cytokine that initiates a cascade of reactions via the upregulation of IL-1 and IL-6 levels resulting in multiple organ injuries. In the present study, MCP treatment significantly upregulated TNF-α, IL-1β, IL-6 and IL-12 cytokines level, while the NAC pretreatment downregulated their levels, suggesting the potential anti-inflammatory properties of NAC in MCP-exposed rats.

Oxidative stress-induced biomolecular damage is closely related to apoptosis related proteins. Bcl-2 family proteins are prime anti-apoptotic members and their over expression effectively prevents free radical-mediated apoptosis [90]. Bcl-2 proteins inhibit activation of caspase-3 and maintain membrane permeability and fluidity. On the other hand, Bax and caspase-3 accelerate apoptosis. In the present study, increased expression of Bax suggests the formation of mitochondrial transition pore leading to the membrane destabilization in MCP-exposed rats. The pretreatment of NAC significantly downregulated Bax, caspase-3 expression and upregulated Bcl-2 levels, and thereby prevented apoptosis in MCP-exposed rats liver tissue.

The histological and electron microscopy assessment of liver tissue also validated the biochemical changes and molecular alterations following MCP exposure. The findings demonstrated significant degenerative and structural changes in the histology and electron microscopy of liver tissues, which are direct shreds of evidence to oxidative damage, apoptosis and inflammations reported in the study. Congestion of the central vein, cell infiltrations and sinusoidal disruptions along the fibrosis observed in the present study can be correlated to the oxidative damage of lipids and proteins. Histological findings of the present study are in agreement with the previous study, which depicts oxidative stress as a culprit for MCP-mediated toxicity in liver tissue [91]. Ultra structural changes like nuclear membrane breakage, change in nuclear shape and chromatin condensation, as seen in our study, are also testimony to MCP-induced cellular damages. Further, the study demonstrated that prior administration of NAC to MCP-exposed rats reduced the histological alternations and the ultra structural changes, as evidenced by the restoration of cellular structures and arrangement of hepatocytes, central veins and nucleus. The prior treatment of NAC significantly reduced the congestion of central vein, cell infiltrations, sinusoidal disruption, fibrosis, inflammation and apoptotic nuclei in liver tissue of MCP-exposed rats. In the present study, histological and ultra structural assessments of hepatic tissue confirmed the ameliorative potential of the NAC oral supplements against MCP-induced toxicity. The findings suggest the anti-oxidative and anti-apoptotic potential of NAC against MCP-induced structural and molecular alterations in the rat liver tissue.

## 5. Conclusions

**Figure 5:**
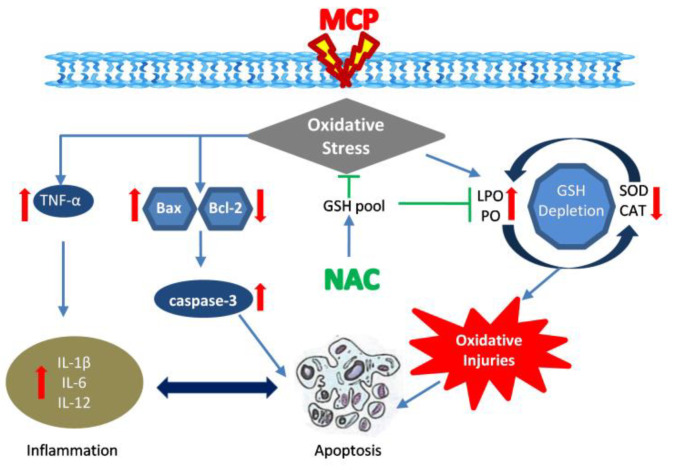
Hypothetical representation of protective potential of NAC against MCP-induced hepatotoxicity in rats.

The findings of the present study suggest that NAC might be effective in ameliorating oxidative stress and structural changes in the hepatic tissue of rats following MCP exposure. The reduction of LPO, protein oxidation and augmentation of antioxidant enzymes are major protective evidence of NAC against MCP exposure. In addition, the results depicted that NAC also attenuates MCP-induced histological and ultra structural anomalies of the liver and down-regulated the apoptotic and inflammatory cascade via regulating Bax, Bcl–2 expressions and TNF-α expressions. The findings suggest that the NAC ameliorates MCP toxicity by assisting GSH synthesis, quenching the free radicals and protecting biomolecular profiles in the tissue (Figure 5). The ameliorative potential of NAC and other potent antioxidants is still obscure, and a more scientific approach via in vivo and in vitro investigation is warranted to identify the molecular mechanism of action and effectiveness of NAC.

## Figures and Tables

**Table 1:** The specific primers sequence used for PCR quantification of mRNA levels.

Gene	Accession #	Direction	Sequence (5′ to 3′)
β-actin	V01217.1	Forward	TTGCCCTAGACTTCGAGAAA
		Reverse	AGACTTACAGTGTGGCCTCC
IL-1β	NM_031512.2	Forward	GGGATGATGACGACCTGCTA
		Reverse	TGTCGTTGCTTGTCTCTCCT
IL-6	NM_012589.2	Forward	AGCCAGAGTCATTCAGAGCA
		Reverse	GGTCTTGGTCCTTAGCCACT
IL-12	NM_022611.1	Forward	GATGCTGGCCAATACACCTG
		Reverse	CAAGTCCGTGTTTCTGTGCA
TNF-α	NM_012675.3	Forward	CATGAGCACGGAAAGCATGA
		Reverse	TAGACAGAAGAGCGTGGTGG

PCR was done for 30 cycles consisting 1 min of each, at 95 °C (denaturation), annealing and 70 °C (elongation). The final extension was done for 10 min at 72 °C. Prepared PCR products were separated using 1.8% agarose gel electrophoresis containing 0.4 μg/mL ethidium bromide. The bands were visualized and photographed using gel documentation system (Bio-Rad Laboratries, Hercules, CA, USA).

**Table 2:** Effects of NAC pretreatment on MCP exposure induced alterations in hepatic antioxidant status of rats.

Group	SOD (U/mg Protein)	Catalase (μmol H_2_ O_2_ Decomposed /min/mg Protein)	GSH (nmol/mg Protein)
Cont	8.73 ± 0.35	50.80 ± 4.82	41.66 ± 4.08
NAC	8.41 ± 0.60	52.17 ± 4.60	43.73 ± 2.77
MCP	5.44 ± 0.51 ^a^ ***	28.20 ± 3.56 ^a^ ***	25.61 ± 2.03 ^a^ ***
NAC + MCP	7.03 ± 0.37 ^b^ ***	40.20 ± 5.07 ^b^ ***	36.94 ± 2.01 ^b^ ***

The values represented are mean ± S.D. (*n* = 5). ^a^ as compared to the control group; ^b^ as compared to MCP-exposed group. *** *p* < 0.001, significantly different.

**Table 3:** Description of frequency assignment of FTIR spectra in rat liver.

Wavenumber (cm^−1^)				Peak Assignment
Control	NAC	MCP	NAC + MCP	
3297	3297	3294	3296	N-H stretch of proteins: mainly amide A
3080	3081	3077	3080	N-H stretch of proteins: mainly amide B
3014	3014	3012	3014	Olefinic = C-H stretch: unsaturated lipids
2959	2961	2952	2957	CH_3_asymmetric stretching: mainly lipids
2925	2926	2921	2923	CH_2_ asymmetrical stretching: mainly lipids
2854	2854	2852	2854	CH_2_symmetric stretch: lipids
1745	1744	1738	1742	Ester C = O stretch: lipids
1652	1653	1649	1652	C = O stretch of proteins: amide I
1541	1541	1537	1540	N-H bend, C-N stretch of proteins: amide II
1456	1458	1451	1456	CH_2_ bend: lipid and protein
1397	1398	1393	1396	COO- symmetric stretch: fatty acids

**Table 4:** Effect of NAC pretreatment on peak area values of FTIR spectra in hepatic tissue of rat exposed to MCP.

Peak Position	Experiment Groups			
	Cont	NAC	MCP	NAC + MCP
3297 cm^−1^	170.33 ± 3.42	169.95 ± 2.96	108.00 ± 4.07 ^a^ ***	163.05 ± 3.17 ^b^ ***
3080 cm^−1^	17.97 ± 1.35	18.44 ± 1.73	12.46 ± 0.93 ^a^ ***	16.73 ± 1.12 ^b^ ***
3014 cm^−1^	2.53 ± 0.35	2.69 ± 0.41	1.77 ± 0.14 ^a^ ***	2.44 ± 0.27 ^b^ ***
2959 cm^−1^	9.49 ± 0.86	9.53 ± 0.94	07.70 ± 1.01 ^a^ *	08.72 ± 0.78 ^b^ *
2925 cm^−1^	14.08 ± 0.61	14.14 ± 0.53	11.25 ± 0.76 ^a^ **	12.58 ± 0.62 ^b^ **
2854 cm^−1^	20.70 ± 0.77	21.07 ± 0.87	14.67 ± 1.12 ^a^ ***	18.88 ± 0.68 ^b^ ***
1745 cm^−1^	01.53 ± 0.15	01.62 ± 0.12	0.93 ± 0.12 ^a^ ***	01.37 ± 0.08 ^b^ ***
1652 cm^−1^	29.96 ± 1.73	29.12 ± 1.23	14.68 ± 1.52 ^a^ ***	25.28 ± 1.28 ^b^ ***
1540 cm^−1^	17.41 ± 1.27	16.77 ± 1.02	09.01 ± 1.36 ^a^ ***	14.89 ± 1.11 ^b^ ***
1456 cm^−1^	04.39 ± 0.38	04.58 ± 0.59	02.38 ± 0.37 ^a^ ***	03.49 ± 0.45 ^b^ ***
1397 cm^−1^	05.69 ± 0.72	05.87 ± 0.44	03.23 ± 0.60 ^a^ ***	04.48 ± 0.19 ^b^ ***

The values represented are mean ± S.D. (N = 3). ^a^ as compared to control; ^b^ as compared to MCP-exposed group. * *p* < 0.05; ** *p* < 0.01; *** *p* < 0.001, significantly different.

**Table 5:** Description of histological alterations and structural assessment in different groups of the study (N = 3).

Parameters	Control	NAC	MCP	NAC + MCP
H and E Stain				
Congestion of central vein	-	-	++	+
Sinusoidal space	-	-	+++	+
Inflammation and infiltrated cells	-	-	+++	+
Apoptotic hepatocytes	-	-	++	+
Pyknotic nuclei	-	-	+++	-
Activated kuffer cells	-	-	++	-
Van Gieson’s Stain				
Focal necrosis plaques	-	-	++	+
Replication of collagen fibers	-	-	+++	+
Fibrotic generation	-	-	++	-
Transmission Electron Microscopy				
Ovulated nucleus	-	-	+++	+
Breaks in nuclear envelope	-	-	+++	-
Hyper chromatic chromatids	-	-	+++	+

Sign indicate none, + indicates mild alterations, ++ indicates moderate alterations, +++ indicates severe alterations.

## Data Availability

The data is contained within the article.
